# Bacterial Endocarditis Caused by *Actinomyces oris*: First Reported Case and Literature Review

**DOI:** 10.1177/2324709620910645

**Published:** 2020-03-05

**Authors:** Chanita Phichaphop, Nopporn Apiwattanakul, Suthep Wanitkun, Sophida Boonsathorn

**Affiliations:** 1 Faculty of Medicine Ramathibodi Hospital, Mahidol University, Bangkok, Thailand

**Keywords:** *Actinomyces* infection, infective endocarditis, culture-negative endocarditis

## Abstract

*Actinomyces* species are gram-positive, facultative anaerobic bacilli. Infection caused by *Actinomyces* species is usually limited to cervicofacial, thoracic, and abdominopelvic regions. Infective endocarditis due to *Actinomyces* species is extremely rare with only 30 reported cases since 1939. We report a case of *Actinomyces oris* endocarditis in a 14-year-old boy who had a 2-week history of dyspnea on exertion without other constitutional signs. Transthoracic echocardiography was suggestive of perforation of the right coronary cusp of aortic valve. No organisms were isolated from blood cultures. The patient underwent surgical valve repair due to deteriorated cardiac function. Valve tissue culture did not initially identify the organism. However, the terminal subculture in a thioglycolate broth grew gram-positive bacilli. The matrix-assisted laser desorption ionization time-of-flight mass spectrometry (MALDI-TOF MS) was compatible with *Actinomyces oris*. After 6 weeks of intravenous ampicillin, the patient remained well with improved cardiac function. We reviewed all reported cases of infective endocarditis caused by *Actinomyces* species, commenting on clinical characteristics and factors associated with unfavorable outcomes in infective endocarditis due to *Actinomyces* species. Although infective endocarditis caused by *Actinomyces* spp is rare, it could be considered in a case of culture-negative endocarditis since the clinical features might be indistinguishable from other bacterial endocarditis. Additionally, MALDI-TOF MS is a useful diagnostic tool for the identification of *Actinomyces* spp to improve the accuracy of diagnosis.

## Introduction

*Actinomyces* species are gram-positive, facultative anaerobic bacilli. They can be part of oral cavity, gastrointestinal tract, and vaginal flora. Infection caused by *Actinomyces* species is usually indolent and is typically limited to cervicofacial, thoracic, and abdominopelvic regions.^1^ Actinomycotic endocarditis is extremely rare. In this article, we describe the first case of infective endocarditis caused by *Actinomyces oris*.

## Case Presentation

A previously healthy 14-year-old boy from the western part of Thailand presented with a 2-week history of dyspnea on exertion. He had no fever or other constitutional symptoms suggestive of infection. He denied history of cardiac diseases, recent dental procedures, or intravenous drug use. Physical examination at the referring hospital was notable for a systolic ejection murmur grade 3/6 at the left upper sternal border. The lungs were clear, and the liver was 3 cm below the right costal margin. Laboratory evaluation revealed a white blood cell count of 16 200/µL with 76% neutrophils, hemoglobin of 13 g/dL, platelet count of 464 000/µL, an erythrocyte sedimentation rate of 7 mm/h, and an anti-streptolysin O titer >400 IU. A chest X-ray revealed evidence of congestive heart failure. In addition to diuretics and inotropic drugs, benzathine penicillin and oral prednisolone were given as presumed acute rheumatic fever. He later developed a high-grade fever without any foci of infection. Meropenem was started empirically without obtaining a blood culture. He did not respond to initial therapy and was referred to our hospital.

**Figure 1.**
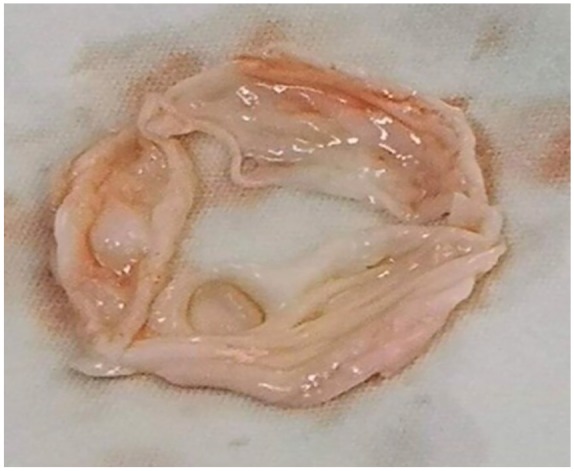
Gross pathology of mitral valve. Circular thinning lesion on the right cusp without perforation is shown.

Physical examination at our hospital revealed an afebrile child with stable vital signs but had gross dental caries. Subcutaneous nodules, Osler’s nodes, Janeway lesions, and splinter hemorrhages were absent. Cardiac examination showed both left and right ventricular heave, normal S1, loud P2, a to-and-fro murmur grade 3/6 at left upper sternal border, and a pansystolic murmur grade 3/6 at apex. Neurological and fundoscopic examinations were unremarkable. Laboratory findings included a white blood cell count of 6700/µL with 83% neutrophils, a hemoglobin level of 12 g/dL, platelet count of 242 000/µL, and an erythrocyte sedimentation rate of 6 mm/h. Urinalysis revealed 0 to 1 white blood cell/high-power field and over 20 red blood cells/high-power field. Chest X-ray showed cardiomegaly with pulmonary congestion. Transthoracic echocardiogram revealed biventricular hypertrophy with an ejection fraction of 49% with evidence of severe aortic valve (AV) regurgitation with a suspected perforation of both the right coronary cusp 5.2 × 5.6 mm and noncoronary cusp 5 × 8 mm, severe mitral valve (MV) regurgitation with an abnormal MV leaflet. No vegetations were seen. These findings suggested infective endocarditis according to the modified Duke criteria.^2,3^ Four sets of blood cultures were obtained, and he was empirically treated with ampicillin/sulbactam (3 g every 6 hours) and gentamicin (120 mg every 8 hours). No organisms were isolated after 5 days of incubation. He subsequently underwent surgical AV repair as indicated by worsening cardiac function. Operative findings revealed severely damaged MV and AV due to restriction and thickened cusps and a circular thinning lesion on the right coronary cusp. However, no vegetation or perforation was noted. The MV was repaired, and the AV was replaced (Figure 1).

**Figure 2.**
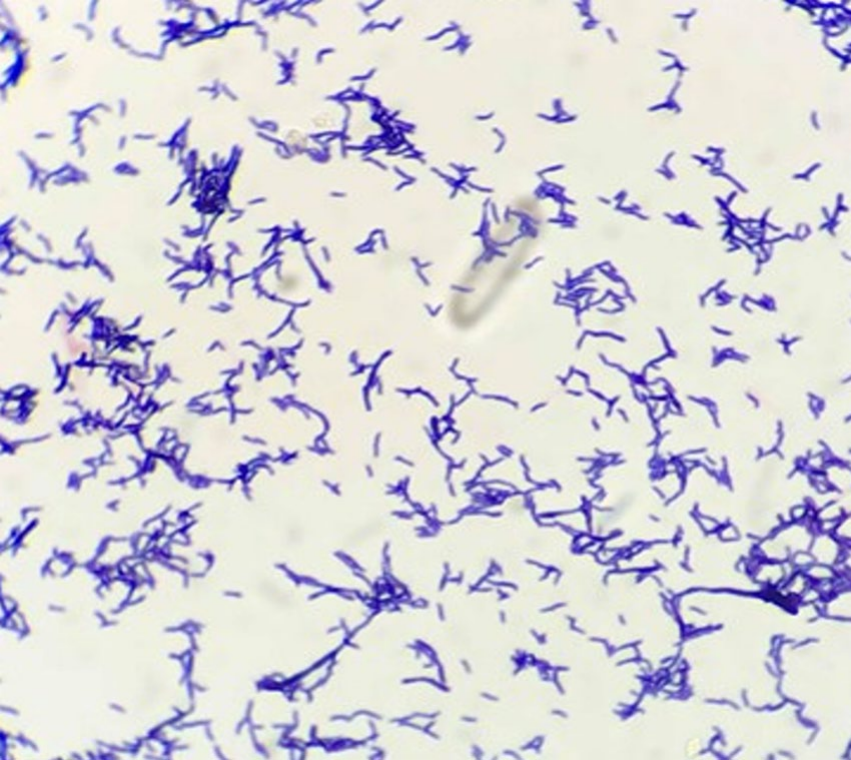
Gram stain of the organism grown from mitral valve tissue culture in thioglycolate broth. Gram stain showed gram-positive bacilli with small branching.

Mitral valve and AV tissues were obtained for aerobic culture and 16s rRNA sequencing, which initially were unable to culture or identify an organism. The histopathologic examination of both valves revealed white myxomatous degeneration and fibrosis without vegetation or perforation, compatible with post-inflammatory valve disease. The terminal subculture in a thioglycolate broth grew gram-positive, small branching bacilli after 120 hours of incubation (Figure 2).

The biochemical tests and matrix-assisted laser desorption ionization time-of-flight mass spectrometry (MALDI-TOF MS) were compatible with *A oris*, with the susceptibility test as shown in Table 1. The patient was diagnosed with *A oris* endocarditis with suspected underlying rheumatic heart disease. Antibiotics were switched to intravenous ampicillin (12 g/day) for 6 weeks. The follow-up echocardiography showed an ejection fraction of 45% with trivial AV regurgitation and mild MV regurgitation. Ampicillin was switched to oral amoxicillin 2 g twice daily for a planned 12-month total course. At the follow-up visit 6 months later, he remained well and improved from functional class IV to II.

**Table 1.** Susceptibility Testing of *Actinomyces oris*.

Drugs	MIC (µg/mL)	Interpretation
Penicillin	0.12	S
Gentamicin	≤2	S
Rifampicin	≤0.5	S
Vancomycin	1	S
Clindamycin	0.5	S
Erythromycin	≤0.25	S
Tetracycline	≤2	S
Linezolid	1	S
Trimethoprim-sulfamethoxazole	>4	R
Daptomycin	>4	R
Ciprofloxacin	4	R

Abbreviations: MIC, minimal inhibitory concentration; S, susceptible; R, resistant.

## Literature Review

Previously reported cases of endocarditis caused by *Actinomyces spp* were searched by using the keywords “actinomyces spp” OR “actinomyces” OR “actinomycotic” AND “infective endocarditis” OR “endocarditis” in PubMed database.

## Discussion

*Actinomyces* species is a gram-positive, filamentous, facultative anaerobic bacilli. Infective endocarditis caused by *Actinomyces* species is rare with only 30 reported cases since 1939. To date, 14 species of *Actinomyces* have been implicated in endocarditis: *Actinomyces bovis, Actinomyces graminis, Actinomyces septicus, Actinomyces muris, Actinomyces israelii, Actinomyces viscosus, Actinomyces meyeri, Actinomyces pyogenes, Actinomyces funkei, Actinomyces odontolyticus, Actinomyces neuii, Actinomyces georgiae, Actinomyces turicensis*, and *Actinomyces naeslundii*. To the best of the authors’ knowledge, this is the first reported case of *A oris* as a causative organism of infective endocarditis.

*Actinomyces oris* is one of the predominant organisms colonizing the oral cavity and plays a role in dental plaque formation. This species previously belonged to the *A naeslundii/A viscosus* group. However, the multilocus sequence analysis based on sequence comparisons for partial gene sequences has further speciated and proposed *A oris* as a new species of *Actinomyces*. Furthermore, a phylogenetic tree based on 16s rRNA gene sequence of the genus *Actinomyces* has clearly showed that *A oris* is genetically different from *A naeslundii* and *A viscosus*.^4-6^ However, it is also possible that *A viscosus* or *A naeslundii* in previous reports might be actually *A oris* as the technology at that time might not be able to differentiate these species.

In a literature review, 31 cases of endocarditis caused by *Actinomyces spp* have been reported since 1939 including our case (Table 2). Of the previous case reports of actinomycotic endocarditis, there was only one pediatric case. The median age was 48 years (34-65 years), and 22 patients (71%) were male. Sixteen patients (52%) had underlying cardiac disease. Seven patients (22.6%) had a history of recent dental procedure or presence of dental caries. Twenty-eight cases (90.3%) involved a native valve. Of these 31 cases, 8 patients (25.8%) required cardiac surgery. The overall mortality associated with actinomycotic endocarditis was 25.8% (8 of 31 patients). Clinical characteristics, treatment, and outcome of patients with *Actinomyces* endocarditis are described in Table 3.

**Table 2.** Summary of 30 Reported Cases Diagnosed With Infective Endocarditis Attributable to *Actinomyces* Species.

Case (Reference)	Year	Age	Sex	Duration of Illness (Months)	Valve(s)	Predisposing Factors	Organism	Therapy	Duration of Treatment (Months)	Outcome
1 (17)	1939	24	Male	1	MV, AV	None	*Actinomyces bovis*	Sulfathiazole	NA	Dead
2 (18)	1945	55	Male	9	MV, AV	Aortic insufficiency, dental caries	*Actinomyces graminis*	None	NA	Dead
3 (19)	1946	39	Male	6 weeks	MV	Cardiac murmur	*Actinomyces septicus*	PCN	10	Survived
4 (20)	1947	37	Male	NA	MV	RHD	*Actinomyces* spp	Sulfathiazole	6	Dead
5 (20)	1947	71	Female	NA	AV	RHD	*Actinomyces* spp	None	NA	Dead
6 (21)	1951	27	Male	2	MV	RHD	*Actinomyces muris*	Chloramphenicol	1	Survived
7 (22)	1962	43	Male	2	MV	RHD, dental caries	*Actinomyces bovis*	PCN	5.5	Survived
8 (23)	1968	6	Male	NA	MV	RHD	*Actinomyces israelii*	PCN	8 days	Dead
9 (24)	1976	70	Male	5	MV	Periodontitis	*Actinomyces viscosus*	PCN	2.5	Survived
10 (25)	1993	65	Male	1	MV, AV	RHD, H/O endocarditis	*Actinomyces israelii*	PCN	7.5	Survived
11 (26)	1996	48	Male	2 weeks	AV	None	*Actinomyces meyeri*	PCN	1.5	Survived
12 (27)	1997	64	Male	1	AV	AS	*Actinomyces pyogenes*	CTX → VAN + AMP + GEN	NA	Dead
13 (15)	1998	81	Male	2-3 weeks	AV	Poor dental hygiene	*Actinomyces viscosus*	Ceftizoxime and CTX	3	Survived
14 (28)	1998	55	Male	NA	MV	None	*Actinomyces meyeri*	AMP/SUL	1.5	Survived
15 (29)	2001	38	Male	2 weeks	MV	None	*Actinomyces viscosus*	VAN + GEN → CTM + PCN	NA	Survived
16 (30)	2002	40	Female	2 weeks	TV	Dental root infection, IVDU, H/O endocarditis	*Actinomyces funkei*	Cefuroxime + RIF + CLN → CTX → CLN	NA	Survived
17 (31)	2005	33	Male	2	TV	IVDU, dental procedure	*Actinomyces odontolyticus*	CTX → PCN + MET	NA	Survived
18 (7)	2005	43	Female	2 weeks	AV	Dental cleaning	*Actinomyces viscosus*	AMP + azithromycin → VAN + GEN + CTX	1	Survived
19 (32)	2007	68	Male	3 weeks	AV	Dental procedure	*Actinomyces neuii*	AMP + GEN + CTX → AMP → doxycycline	12	Survived
20 (33)	2007	34	Male	NA	MV	RHD	*Actinomyces* spp	NA	NA	Dead
21 (34)	2008	27	Female	2 days	EV	IVDU, H/O endocarditis	*Actinomyces israelii*	Unclear antibiotics, surgery	NA	NA
22 (35)	2008	46	Male	1	MV	None	*Actinomyces georgiae*	PCN → CTX → AMP	8.5	Survived
23 (14)	2010	66	Male	2	PAV	Aortic insufficiency	*Actinomyces neuii*	PCN + MER + ERY → amoxicillin	12	Survived
24 (36)	2010	87	Male	2	MV	Dental cleaning	*Actinomyces israelii*	PCN	7.5	Survived
25 (37)	2013	49	Male	NA	TV	IVDU	*Actinomyces* spp	Van → CTX → ciprofloxacin + MET	NA	Survived
26 (38)	2014	67	Male	6 weeks	PAV	Prosthetics, dental cleaning	*Actinomyces naeslundii*	CTX	1.5	Dead
27 (39)	2015	30	Female	1 week	EV	None	*Actinomyces turicensis*	PCN → CTX	2	Survived
28 (40)	2015	51	Female	2	PAV	Prosthetics, dental caries	*Actinomyces naeslundii*	VAN + CTX → CTX → ERT → amoxicillin	12	Survived
29 (41)	2018	55	Female	8	MV, AV	HOCM with LVOT	*Actinomyces israelii*	PCN	11	Survived
30 (13)	2019	61	Male	1 week	MV, AV	H/O MV endocarditis	*Actinomyces neuii*	VAN + PIP/TAZ → AMP + GEN → AMP → doxycycline	12	Survived
This case	2019	13	Male	2 weeks	MV, AV	Dental caries, probable RHD	*Actinomyces oris*	AMP/SUL → AMP → amoxicillin	12	Survived

Abbreviations: NA, not applicable; H/O, history of; RHD, rheumatic heart disease; IVDU, intravenous drug use; MV, mitral valve; AV, aortic valve; TV, tricuspid valve; PAV, prosthetic aortic valve; EV, eustachian valve; HOCM, hypertrophic cardiomyopathy; LVOT, left ventricular outlet obstruction; PCN, penicillin; AMP, ampicillin; CTX, ceftriaxone; CTM, cefotaxime; VAN, vancomycin; ERT, ertapenem; MER, meropenem; GEN, gentamicin; AMP/SUL, ampicillin/sulbactam; CLN, clindamycin; ERY, erythromycin; RIF, rifampicin; MET, metronidazole; PIP/TAZ, piperacillin/tazobactam.

**Table 3.** Clinical Characteristics, Treatment, and Outcome of Endocarditis Cases Caused by *Actinomyces* Species.

Clinical Characteristics	N (%)
Age, years, (range)	48 (34-65)
Sex (male)	22 (71)
Underlying cardiac disease	16 (52)
History of recent dental procedures or presence of dental caries	7 (22.6)
Native valve	28 (90.3)
Mitral valve	11 (35.5)
Aortic valve	6 (19.4)
Mitral and aortic valve	6 (19.4)
Eustachian valve	2 (6.5)
Prosthetic valve	3 (9.7)
Treatment with non–β-lactams antibiotics	6 (19.4)
Required surgery	8 (25.8)
Duration of treatment (months) (range)	1-12
Death	8 (26.7)

Similar to the present case, most of the patients in this review presented with subacute or chronic endocarditis that usually involved native heart valves. Predisposing factors for actinomycotic endocarditis include periodontal diseases or dental procedures in association with a preexisting cardiac valvular defect. Our patient might have had underlying rheumatic heart disease that he had not been aware of. This is a known risk factor for infective endocarditis. Additionally, the pathological findings from the MV and AV were suggestive of post-inflammatory change, which can be seen in rheumatic heart disease. Furthermore, the presence of dental caries, in this case, might be an attributable factor for developing infective endocarditis since *Actinomyces* species habitually colonize in the oral cavity.

The diagnosis of actinomycotic endocarditis primarily depends on the identification of *Actinomyces* species from blood cultures, which may be recognized within 5 to 7 days. However, the cultures should be held for up to 4 weeks to improve the yield of diagnosis. Moreover, blood cultures may fail to identify the organism since these facultative anaerobes require special specimen handling with minimal exposure to oxygen and a need for a CO_2_-enriched environments.^7,8^ The definitive diagnosis of *Actinomyces* spp has always been challenging. Over the past decade, 16s rRNA sequencing has been widely used for bacterial identification and the discovery of novel bacteria, especially uncultivable or slow-growing bacteria.^9^ This method has led to the classification and identification of *Actinomyces* spp, differentiating *Actinomyces* spp from other gram-positive anaerobic bacilli.^10^ However, accurate identification of certain species of actinomycosis is still problematic. MALDI-TOF MS has emerged as a rapid and effective method for bacterial identification with the ability to speciate closely related organisms.^11,12^ A previous study has demonstrated the performance of MALDI-TOF MS in identification of endocarditis due to *A neuii*.^13^ As in this case, MALDI-TOF MS was used to confirm the etiologic organism in subacute endocarditis.

The choice and optimal duration of antibiotics in actinomycotic endocarditis remains unclear. *Actinomyces* species are generally susceptible to β-lactam antibiotics. Penicillin or cephalosporins have been considered to be first-line agents for the treatment of actinomycosis. According to previous reports, most patients with endocarditis tended to receive high doses and prolonged antibiotic therapy.^14,15^ In our literature review, duration of antibiotic therapy ranged from 1 to 12 months. Alternative agents, including chloramphenicol, erythromycin, clindamycin, doxycycline, or vancomycin, have been shown in vitro to be active against these organisms.^16^ In the present case, the patient was successfully treated with 6 weeks of intravenous ampicillin followed by oral amoxicillin for a planned 12-month course.

In conclusion, we describe a case of native valve *A oris* endocarditis that was successfully treated with intravenous ampicillin and oral amoxicillin and surgical valve replacement. Although infective endocarditis caused by *Actinomyces* spp is rare, it could be considered in a case of culture-negative endocarditis since the clinical features might be indistinguishable from other bacterial endocarditis. Additionally, MALDI-TOF MS could be a useful diagnostic tool for the identification of *Actinomyces* spp to improve the accuracy of speciation and diagnosis.
